# Prevalence and Risk of Mild Cognitive Impairment Among Chinese Adults Aged 60 Years and Above: A Cross-Sectional Study Based on National Data

**DOI:** 10.62641/aep.v54i4.2096

**Published:** 2026-08-15

**Authors:** Qingpan Wen, Xinli Zhou, Changyi Xie, Hongying Pan, Huan Zhou, Marcin Białas, Bifen Dai, Jiepeng Lei

**Affiliations:** ^1^Faculty of Physical Education, Gdansk University of Physical Education and Sport, 80-336 Gdansk, Poland; ^2^Department of Physical Education, Chengdu Sport University, 610041 Chengdu, Sichuan, China; ^3^Department of Psychiatry, The Affiliated Mental Health Center of Kunming Medical University, 650224 Kunming, Yunnan, China

**Keywords:** mild cognitive impairment, prevalence, risk factors, depression score, ageing

## Abstract

**Background:**

This study conducted a cross-sectional analysis to assess the prevalence of mild cognitive impairment (MCI) among older adults in China and to identify its associated risk factors, with the aim of informing the development of early intervention strategies.

**Methods:**

This cross-sectional study used data collected in 2018 (Wave 4). The initial survey sample comprised 19,744 individuals, of whom 4773 formed the valid sample for analysis. After screening, the study ultimately selected 694 individuals who met all the inclusion criteria. MCI was defined according to the criteria for age-associated cognitive decline (AACD). Sociodemographic information, past comorbidities, and lifestyle were assessed.

**Results:**

The prevalence of MCI was 14.54%, with 694 individuals diagnosed with MCI. An elevated Center for Epidemiologic Studies Depression (CESD) score was identified as a significant risk factor for MCI (OR = 1.6, 95% CI: 1.3–1.9; *p* < 0.001). Living in rural areas (odds ratios (OR) = 2.0, 95% confidence interval (CI): 1.6–2.6; *p* < 0.001) and napping (OR = 1.3, 95% CI: 1.1–1.6; *p* = 0.002) were also significant risk factors for MCI. Additionally, gender, memory level, and nighttime sleep were each associated with a 1.2-fold increase in MCI risk (OR = 1.2 for all; *p* range: 0.001–0.047). Correlation analysis indicated that both age and the current CESD score were significantly negatively correlated with the total cognitive score (*p* < 0.001).

**Conclusions:**

Among Chinese older adults living in the community, CESD score, napping, nighttime sleep, being female, and memory level were identified as independent risk factors for MCI. These findings underscore the importance of implementing screening and preventive strategies to mitigate MCI risk in this demographic.

## Introduction

China is facing a profound challenge of population ageing and currently has the largest population of older adults in the world [1]. According to data from the seventh national census of China conducted in 2020, the population aged 60 and above reached 264 million, accounting for 18.70% of the total population [2]. This trend reflects a broader global phenomenon, as population ageing is widely recognised as a major transformation in the 21st century [3]. It is projected that the global population aged 65 and above will reach nearly one billion by 2030 [4]. Due to the large and rapidly growing ageing population, the proportion of expenditures on pensions and healthcare relative to gross domestic product is expected to increase from 7.33% in 2015 to 26.24% by 2050 [5]. With the acceleration of population ageing in China, the incidence of Alzheimer’s disease (AD) has also been increasing. Cognitive disorders not only diminish the quality of life of older adults but also impose substantial economic and caregiving burdens on families and society [6, 7, 8]. Among the health-related risk factors contributing to disability in the older adults, the prevalence of mild cognitive impairment (MCI) and its associated determinants have received extensive attention in the clinical field.

MCI is considered a transitional stage between normal ageing and dementia and is generally regarded as an early manifestation of AD [9]. Previous research has shown that approximately 10–15% of patients with MCI progress to AD each year [10]. Currently, most forms of dementia, including AD, lack effective pharmacological treatments [11]. However, MCI may represent a potentially reversible state and constitutes a critical transitional stage between normal cognitive ageing and dementia. Early detection and intervention for MCI may therefore help restore patients to a normal state and are of great significance in delaying or reversing the course of AD. Consequently, identifying the risk factors for cognitive impairment is essential for developing effective strategies to delay the progression of dementia.

Existing studies have indicated that the occurrence of MCI is closely associated with factors such as gender, age, educational level, apolipoprotein E alleles, smoking, depressive mood, and chronic diseases [12, 13, 14, 15]. A meta-analysis shows that the prevalence of cognitive impairment among Chinese individuals aged over 55 years is 15.4% [16]. Moreover, different living environments also affect the prevalence of MCI. For instance, in an urban area (Huangpu District, Shanghai), the prevalence of MCI among 802 surveyed older adults was 31.80% [17], whereas in a rural area (Shanxi Province), the prevalence among 1064 older adults was 17.6% [18]. This study used data from the China Health and Retirement Longitudinal Study (CHARLS). The survey sample is a nationally representative survey covering around 10,000 households from 150 counties (districts) and 450 villages (communities) nationwide across China. The survey includes approximately 17,000 middle-aged and older adults, providing high-quality microdata on individuals and households.

In this cross-sectional study, data from 19,744 older adults were initially considered. The primary objective was to assess the prevalence of MCI and to identify its associated risk factors. In addition, self-reported data on depression and sleep quality were analysed to further explore the association between age and current cognitive function.

## Methods

### Study Population

The CHARLS aims to provide a data foundation for research on China’s ageing population and to support interdisciplinary studies by collecting high-quality microdata. The survey includes families and individuals aged 45 years and older. The CHARLS national baseline survey was conducted in 2011 using a multi-stage probability proportional-to-size sampling method. The survey sample covers 28 provinces across the country (150 counties and 450 villages), involving approximately 10,000 households and over 17,000 individuals. As a continuous project, CHARLS conducts follow-up visits every 2 to 3 years and uses computer-assisted personal interviewing to perform face-to-face household interviews.

The survey content includes basic demographic information of the respondents and their families, transfer payments among family members, employment, income, respondents’ health status, expenditure, medical care and insurance, and assets. In addition, the CHARLS project includes 13 physical measurements and the collection of blood samples. To date, five waves of data have been publicly released, including the national baseline survey in 2011 (Wave 1) and follow-up surveys in 2013 (Wave 2), 2015 (Wave 3), 2018 (Wave 4), and 2020 (Wave 5). Detailed information about CHARLS has been reported in previous literature [19]. The CHARLS dataset can be downloaded from the CHARLS homepage at pku.edu.cn. This study was conducted in accordance with the ethical principles of the Declaration of Helsinki and is reported following the Strengthening the Reporting of Observational Studies in Epidemiology (STROBE) guidelines. As this study is a secondary analysis based on de-identified publicly available CHARLS data, it does not involve personal identity information and poses no additional risks to participants. The CHARLS project was approved by the Biomedical Ethics Committee of Peking University (Approval Number: IRB00001052-11015), and all participants provided written informed consent during the original data collection.

**Fig. 1.**
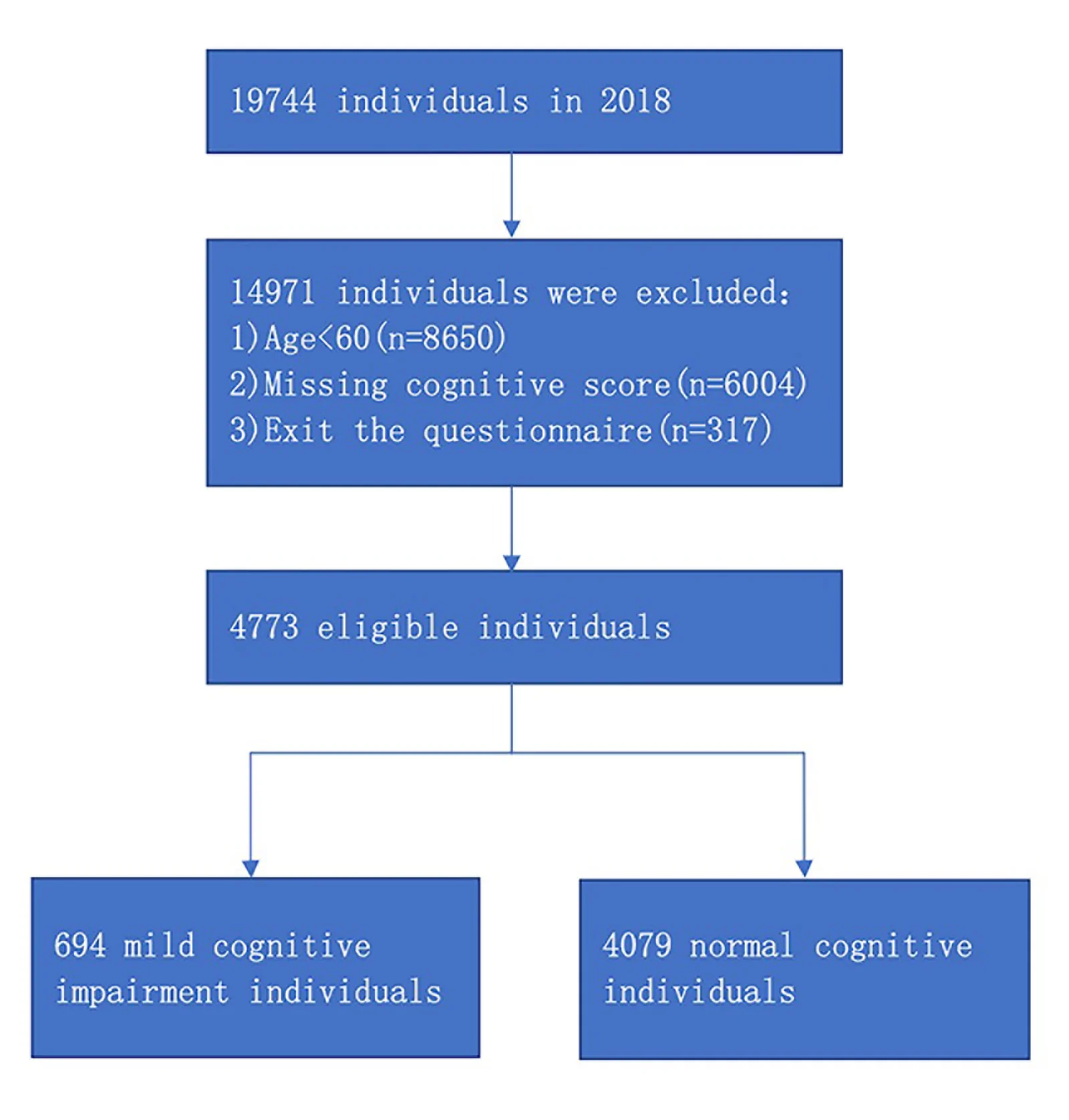
**Flowchart of the sample selection process**.

Data from the 2018 (Wave 4) were used in the present study. Among the initial 19,744 samples, we excluded 8650 individuals under the age of 60 years, 6004 individuals with missing cognitive scores, and 317 individuals who withdrew from the questionnaire survey. Ultimately, 4773 samples were included, consisting of 4079 individuals with normal cognition and 694 individuals with MCI. The specific sample selection process is shown in Fig. 1.

### Measurement of Cognitive Function

This study adopted the standardized methods used in the Health and Retirement Study (HRS) to assess cognitive function [20]. This study is based on the CHARLS data and evaluated in reference to the standardized scoring system of HRS. The cognitive assessment comprised four core components: orientation, computation, memory, and drawing, with a total score of 31 points. This method has been adopted by other related studies [21, 22]. All participants underwent face-to-face assessments covering the four dimensions of cognitive function, including orientation, computation, memory, and drawing. The assessment of orientation and calculation ability referred to the telephone interview for cognitive status scale. The orientation test included the year, month, date, day of the week, and current season, with each correct answer earning 1 point, for a maximum score of 5 points. In the calculation ability test, participants were required to subtract 7 from 100 five consecutive times, and each correct answer earned 1 point. Memory was evaluated using a word recall task. The experimenter randomly read 10 words, and the number of words immediately recalled by the participant was recorded to assess immediate memory ability. Delayed recall ability was then evaluated again after participants completed the depression scale questionnaire, calculation test, and drawing test. The total memory score was the sum of the immediate and delayed recall scores, with a maximum of 20 points, each word counting as 1 point. In the drawing ability test, researchers showed participants a figure composed of two overlapping five-pointed stars and asked them to copy it. If the drawing was accurate, they earned 1 point. Ultimately, the total cognitive score consisted of orientation (5 points), computation (5 points), memory (20 points), and drawing (1 point), with a total score of 31 points.

### Diagnosis of MCI

In this study, we defined MCI using age-associated cognitive decline (AACD), which is at least 1 standard deviation (SD) below the age standard [23, 24]. Participants aged 60 years and older were grouped at 5-year intervals. Participants in each age group who met the AACD criteria were classified as having MCI. In the cross-sectional analysis, this final sample included 694 individuals with MCI and 4079 cognitively normal individuals.

### General Evaluation

Based on prior research, we incorporated sociodemographic characteristics, lifestyle factors, comorbidities, depression scores, memory status, and overall health status into the analysis [25, 26, 27, 28]. Sociodemographic variables included age, gender, place of residence (urban and rural), educational level (illiterate, primary school, junior high school, senior high school/vocational high school, college or above), average family income, and marital status (married, separated, unmarried/divorced/widowed). Lifestyle factors included past or current smoking and alcohol consumption, napping, and nighttime sleep. We also considered the presence of fourteen common comorbid conditions: cancer, chronic pulmonary disease, cardiovascular disease, stroke, mood or mental disorders, arthritis, dyslipidaemia, liver disease, kidney disease, gastrointestinal disorders, asthma, memory-related disorders, hypertension, and hyperglycemia. Information on disease history in CHARLS is mainly based on self-reports, with the prerequisite that the condition had been diagnosed by a doctor. This study employed the 10-item Center for Epidemiologic Studies Depression (CESD)-10 scale, with each item scored from 0 to 3 and a total score ranging from 0 to 30. A score of ≥10 was used as the cut-off value for potential depressive risk [29]. Additionally, this scale has demonstrated good reliability (Cronbach’s α = 0.815) among middle-aged and older adults in China [30]. Memory status and general health status were evaluated through self-reported responses to the question: “Would you rate your memory/health as excellent, very good, good, fair, poor, or very poor?”

### Statistical Analysis

The main outcome of this study was the risk of MCI. Data collected in 2018 (Wave 4) from the CHARLS were used, with a sample size of 19,744. Demographic characteristics and medical history were described using descriptive statistical methods. Categorical variables were presented as frequencies and percentages (n, %), and differences between groups were compared using the χ^2^ test. Before conducting multivariate logistic regression analysis, multicollinearity among independent variables was assessed using the variance inflation factor (VIF). Model goodness of fit was evaluated using Nagelkerke’s pseudo-R^2^. A multivariate logistic regression model was used to identify risk factors for MCI, with the presence or absence of MCI as the dependent variable and covariates including gender, place of residence, and education level. The results were expressed as odds ratios (OR) with 95% confidence interval (95% CI). For continuous variables (such as depression scores), normality tests were first performed before correlation analysis. If the data followed a normal distribution, the Pearson correlation coefficient was used to analyse the correlation; otherwise, Kendall’s tau-b correlation coefficient was applied. All statistical analyses were performed using SPSS 26.0 (SPSS Inc., Chicago, Illinois, USA). A two-tailed *p* value < 0.05 was considered statistically significant.

## Results

### The Prevalence of MCI

A total of 4079 older adults had normal cognition, and 694 older adults had MCI. The overall prevalence of MCI was 14.54%. The prevalence of MCI was significantly higher among female older adults (19.01%) than among male older adults (11.34%) (*p* < 0.001). The prevalence of MCI significantly decreased with increasing educational level (*p* < 0.001), with a prevalence of 0.93% for those with more than high school education and 30.86% for those with less than elementary education. The prevalence of MCI also significantly decreased with improved memory level (*p* < 0.001), with a prevalence of 6.06% among those with excellent memory and 23.31% among those with poor memory. The prevalence of MCI among older adults living in rural areas (18.63%) was significantly higher than that among those living in urban areas (6.31%) (*p* < 0.001). In addition, marital status, health status, alcohol status, napping, nighttime sleep, and CESD score were significantly associated with the prevalence of MCI. In contrast, age range, smoking status, and co-morbidities showed no statistically significant association with MCI prevalence (Table 1, **Supplementary Table 1**).

**Table 1.** **Prevalence of diseases among older adults stratified by sociodemographic and baseline variables**.

Variables			MCI group (n)	Non-MCI group (n)	Prevalence (%)	χ^2^	*p*-value
Total			694	4079	14.54		
Gender						54.99	< 0.001
	Male		315	2464	11.34		
	Female		379	1615	19.01		
	Age group (years)					5.25	0.263
	60–64		250	1475	14.49		
	65–69		204	1278	13.77		
	70–74		144	746	16.18		
	75–79		72	387	15.69		
	≥ 80		24	193	11.06		
	Education					514.48	< 0.001
	Less than elementary		475	1064	30.86		
	Elementary		142	1204	10.55		
	Middle school		76	1705	4.27		
	More than high school		1	106	0.93		
	Marital status					34.00	< 0.001
	Never		2	15	11.76		
	Married with spouse present		512	3370	13.19		
	Married not living with spouse		29	143	16.86		
	Separated/divorced/widowed		151	551	21.51		
	Residence					129.14	< 0.001
	Urban		100	1448	6.31		
	Rural		594	2595	18.63		
	Memory level					96.24	< 0.001
Excellent			2	31	6.06		
	Very good		34	221	13.33		
	Good		53	355	12.99		
	Fair		336	2587	11.50		
	Poor		269	885	23.31		
	Health status					41.83	< 0.001
	Very good		72	438	14.12		
	Good		72	536	11.84		
	Fair		317	2183	12.68		
	Poor		191	724	20.87		
	Very poor		42	198	17.5		
	Smoking status					0.056	0.814
	Yes		198	1146	14.73		
	No		496	2933	14.46		
Alcohol status						23.248	< 0.001
	Yes		215	1658	11.48		
	No		479	2421	16.52		
Napping						18.525	< 0.001
	Yes		405	2723	12.95		
	No		289	1356	17.57		
Nighttime sleep						45.712	< 0.001
	<7 hours		389	2254	14.72		
	7–8 hours		214	1565	12.03		
	>8 hours		91	260	25.93		
CESD score						57.557	< 0.001
	<10 scores		337	2599	11.48		
	≥10 scores		357	1480	19.43		
Co-morbidities							
	Hypertension					2.806	0.094
		Yes	66	477	12.15		
		No	628	3602	14.85		
	Dyslipidaemia					0.4	0.529
		Yes	68	432	13.6		
		No	626	3647	14.65		
	Hyperglycaemia					0.884	0.347
		Yes	43	217	16.54		
		No	651	3862	14.42		
	Cancer					0.089	0.765
		Yes	10	65	13.33		
		No	684	4014	14.56		
	Chronic lung disease					0.062	0.803
		Yes	37	227	14.02		
		No	657	3852	14.57		
	Hepatic disease					0.48	0.488
		Yes	22	151	12.72		
		No	672	3928	14.61		
	Heart disease					0.007	0.934
		Yes	55	327	14.40		
		No	639	3752	14.55		
	Stroke					0.517	0.472
		Yes	43	225	16.04		
		No	651	3854	14.45		
	Kidney disease					1.13	0.288
		Yes	36	175	17.06		
		No	658	3904	14.42		
	Digestive system disease					2.204	0.138
		Yes	60	288	17.24		
		No	634	3791	14.33		
	Emotional and mental disorders					0.001	0.969
		Yes	4	24	14.29		
		No	690	4055	14.54		
	Memory-related disease					2.025	0.155
		Yes	1	1	50.00		
		No	693	4078	14.53		
	Arthritis					0.582	0.446
		Yes	53	279	15.96		
		No	641	3800	14.43		
	Asthma					0.053	0.817
		Yes	16	100	13.79		
		No	678	3979	14.85		

MCI, mild cognitive impairment; CESD, Center for Epidemiological Studies Depression; χ^2^, Chi-square statistic.

### Diagnosis of Multicollinearity and Evaluation of Regression Models

Before constructing the multiple logistic regression model, the VIF was used to test for multicollinearity among the independent variables. Generally, a VIF value < 10 indicates no serious multicollinearity problem. In this study, all VIF values of the independent variables were below 2, suggesting that there was no significant multicollinearity among the independent variables. The overall goodness of fit of the binary logistic regression model was evaluated using Nagelkerke R^2^, which was 0.35, indicating that the model had moderate explanatory power (Table 2, **Supplementary Table 2**).

**Table 2.** **Diagnosis of VIF**.

Variable	VIF	Tolerance
Gender	1.320	0.758
Education	1.203	0.831
Marital status	1.041	0.961
Residence	1.163	0.860
Memory level	1.124	0.890
Health status	1.162	0.861
Alcohol status	1.207	0.828
Napping	1.018	0.982
Nighttime sleep	1.052	0.950
CESD score	1.121	0.892

VIF, Variance Inflation Factor; CESD, Center for Epidemiological Studies Depression.

### Risk Factors for MCI

This study was based on the classification criteria for each variable (Table 1). Our multivariate analysis revealed that residence was a significant risk factor for MCI (OR = 2.0, 95% CI: 1.6–2.6; *p* < 0.001). The CESD score was associated with a 1.6-fold increased risk of MCI (OR = 1.6, 95% CI: 1.3–1.9). Additionally, napping was associated with a 1.3-fold increased risk of MCI (OR = 1.3, 95% CI: 1.1–1.6; *p* = 0.002). Gender, memory level, and nighttime sleep were each associated with a 1.2-fold increased risk of MCI (OR = 1.2, 95% CI: 1.0–1.5, *p* = 0.047; OR = 1.2, 95% CI: 1.0–1.3, *p* = 0.008; OR = 1.2, 95% CI: 1.1–1.4, *p* = 0.001), respectively. In contrast, health status and alcohol status showed no statistically significant association with the risk of MCI, with OR values of 1.0 (95% CI: 0.9–1.1; *p* = 0.864) and 1.1 (95% CI: 0.9–1.4; *p* = 0.233), respectively. Moreover, higher educational level was significantly associated with a reduced risk of MCI (Table 3).

**Table 3.** ** Multivariate logistic regression analysis of MCI risk among adults over 60 years old**.

Variables	β	S.E.	χ^2^	OR	95% CI	*p*-value
Gender	0.197	0.099	3.952	1.2	1.0–1.5	0.047
Education	–1.042	0.064	263.714	0.4	0.3–0.4	< 0.001
Residence	0.706	0.121	34.202	2.0	1.6–2.6	< 0.001
Memory level	0.164	0.062	6.956	1.2	1.0–1.3	0.008
Health status	–0.008	0.048	0.029	1.0	0.9–1.1	0.864
Alcohol status	0.123	0.103	1.425	1.1	0.9–1.4	0.233
Napping	0.280	0.091	9.571	1.3	1.1–1.6	0.002
Nighttime sleep	0.219	0.068	10.257	1.2	1.1–1.4	< 0.001
CESD score	0.442	0.093	22.716	1.6	1.3–1.9	< 0.001

MCI, mild cognitive impairment; CESD, Center for Epidemiological Studies Depression; β, Beta Coefficient; S.E., Standard Error; χ^2^, Chi-square statistic; OR, Odds Ratio; CI, Confidence Interval.

Correlation analysis showed that age was negatively correlated with the total cognitive score (*p* < 0.001), and with cognitive domain scores, including memory (*p* < 0.001), drawing (*p* = 0.016), and orientation (*p* = 0.046). The CESD score was negatively correlated with the total cognitive score (*p* < 0.001) and with cognitive domain scores (computation, orientation, memory, and drawing) (*p* < 0.001). Nighttime sleep was not significantly correlated with the total cognitive score or cognitive domain scores (*p* > 0.05) (Table 4).

**Table 4.** **Correlation between age, depressive score, nighttime sleep, and cognitive function domain scores**.

		Total score	Computation	Orientation	Memory	Drawing
Age						
	CC	–0.072**	–0.009	–0.022*	–0.088**	–0.029*
	*p*	< 0.001	0.408	0.046	< 0.001	0.016
CESD score						
	CC	–0.095**	–0.082**	–0.081**	–0.074**	–0.061**
	*p*	< 0.001	< 0.001	< 0.001	< 0.001	< 0.001
Nighttime sleep						
	CC	0.000	0.022	–0.003	–0.007	0.004
	*p*	0.981	0.056	0.786	0.519	0.722

CESD, Center for Epidemiological Studies Depression; CC, Correlation Coefficient. Significance levels: * *p* < 0.05; **p < 0.01.

## Discussion

This study analysed 4773 older adults aged 60 and above using the CHARLS database. The results showed that the overall prevalence of MCI in this population was 14.54% (n = 694). Our findings indicate that the prevalence of MCI among older adults is associated with age and depression scores, highlighting the importance of screening for MCI in older adults and implementing strategies to reduce the risk of MCI.

The innovation of this study mainly lies in the following three aspects. First, based on the large-scale representative data from the CHARLS database, the sample in this study is nationally representative of the cognitive health status of the population aged 60 years and above in China, addressing the limitation of insufficient representativeness in previous regional studies. Second, the AACD criteria were adopted to define MCI, which are suitable for large-scale epidemiological surveys and allow efficient collection of cognitive function data among older adults. Finally, this study provides clear empirical evidence for the formulation of targeted national public health intervention strategies and resource allocation plans. Its value lies in clearly revealing the nationwide prevalence of MCI among older adults and its associated influencing factors.

The prevalence of MCI in this study (14.54%) is highly consistent with findings from a global meta-analysis (15.56%) [31]. This consistency is crucial because it suggests that the study population is representative of the global situation in terms of MCI prevalence. This broadens the application of the research findings and enhances their generalisability. However, the prevalence reported in this study is lower than estimates from Asian populations (19.8%) and other regions, including Europe (18%) and North America (21.6%) [32]. These variations in global MCI prevalence may be attributed to differences in disease occurrence influenced by education level, living conditions, and lifestyle factors [13, 32, 33, 34]. Furthermore, heterogeneity in research methods across studies, including the use of different diagnostic criteria, may also affect the results. Nevertheless, given its large-scale, nationally representative sample and standardized assessment protocols, this study provides robust and reliable evidence regarding the burden of MCI among older adults.

The prevalence of MCI is positively associated with age [25], which is consistent with the findings of the present study. Differences in reported prevalence may be attributed to differences in the age composition of study populations and the diagnostic criteria used at the time of data collection. However, we observed a notable phenomenon: the prevalence of MCI in the ≥80-year-old age group was lower than that in each of the younger age groups, although the differences between groups did not reach statistical significance. This observation appears to contradict the widely accepted view that age is the most important risk factor for MCI. We speculate that this may, on the one hand, reflect a “healthy survivor bias” in the sample, indicating that participants in the oldest age group represent a relatively healthier subset of the population; on the other hand, it may be related to the age grouping criteria or the sensitivity of the measurement tools used in the older adults. Therefore, caution should be exercised when interpreting the prevalence of MCI among older adults, and the results of this study may better reflect the characteristics of “healthy surviving” older adults living in the community. Our univariate analysis revealed that MCI prevalence did not differ significantly across age groups among older adults. Consistent with previous epidemiological studies in China, factors such as attentional decline [35], female sex [36], advanced age, low educational attainment [37, 38], and residence in rural areas have been identified as associated risk factors [39]. In the present study, female older adults in the younger age group (aged 60 years and above) exhibited a higher prevalence of MCI (19.01%), which partially aligns with findings of earlier research.

Factors associated with MCI were identified using multivariate logistic regression. Among them, older adults living in rural areas emerged as the most significant risk factor (OR = 2.0), underscoring the critical impact of geographic disparities on cognitive health. Depressive symptoms showed a strong positive association, with the odds of MCI increasing by 1.6 times for each one-point increase in the CESD score. This result is consistent with multiple studies that have explored the biological mechanisms linking depression to cognitive decline [40, 41, 42].

Higher education level demonstrated a strong protective effect against MCI (OR = 0.4), reinforcing the long-term protective role of early-life cognitive engagement. With regard to lifestyle factors, both napping (OR = 1.3) and memory level and nighttime sleep (OR = 1.2) were identified as independent risk factors, highlighting the potential benefits of targeted behavioural interventions. Notably, the effect of age was attenuated in the multivariate model, indicating that age-related cognitive decline may largely stem from potentially intervenable factors, including education and mental health.

In the correlation analysis, a significant inverse relationship was observed between depression score and total cognitive score, indicating that higher depression scores are associated with poorer cognitive performance. In the multiple regression analysis, although age was not identified as an established independent risk factor for MCI, it remained negatively associated with cognitive scores among older adults. The prevalence of cognitive impairment tends to increase with advancing age, and this trend appears to be more pronounced in female [43]. The ageing process involves progressive structural and functional alterations in the brain, most notably significant hippocampal atrophy [44, 45]. Within the ageing hippocampus, multiple neurobiological alterations occur, including elevated oxidative stress and neuroinflammation, dysregulation of intracellular signalling pathways and gene expression, as well as reduced neurogenesis and synaptic plasticity. These changes are closely linked to age-related cognitive decline [46]. Cognitive performance is likely influenced by a range of factors, including educational attainment, occupational background, engagement in physical labour, participation in social activities, exercise habits, tea consumption, sleep quality, dietary patterns, and depression symptoms [38, 39, 47].

The significance of this study lies in the systematic identification of a range of modifiable targets for MCI. Among the factors identified, with the exception of gender, most factors, including educational attainment, residential setting, depressive symptoms, and sleep habits, are potentially modifiable. These findings provide a robust scientific basis for developing targeted public health interventions. For example, enhancing access to education among older adults in rural areas, implementing routine mental health screening and early intervention programs, and promoting healthy sleep hygiene practices may help delay or prevent the onset of MCI.

This study has several limitations. First, its cross-sectional design allows the identification of associations between variables but precludes causal inference. Second, the AACD criteria adopted in this study, although useful as a population-based screening tool for early cognitive decline, have limited specificity and may be influenced by educational level, potentially leading to false positive results. Third, this study relied on self-reported data, which may introduce reporting bias in both the exclusion and inclusion groups. Fourth, the CHARLS database does not include detailed information on key confounding variables, such as vascular risk factors and medication use, which may limit a more comprehensive evaluation of cognitive influencing factors.

## Conclusions

In summary, among Chinese older adults living in the community, residence, CESD score, napping, nighttime sleep, being female, and memory level are independent risk factors for MCI. Higher education level may significantly reduce the risk of MCI. There is a certain degree of correlation between the total cognitive score, age, and depression scores. In the Chinese older adults, those living in rural areas, with a lower educational level, and being female are more prone to cognitive impairment.

## Availability of Data and Materials

The datasets used and/or analyzed during the current study are available from the corresponding authors on reasonable request.

## Supplementary Material

Supplementary material associated with this article can be found, in the online version, at https://doi.org/10.62641/aep.v54i4.2096.
