# Supplementary material for: Prevalence and Risk of Mild Cognitive Impairment Among Chinese Adults Aged 60 Years and Above: A Cross-Sectional Study Based on National Data
Source: Actas Esp Psiquiatr. 2026 Aug 15;54(4):971–81. doi: 10.62641/aep.v54i4.2096 (PMC13574534; doi:10.62641/aep.v54i4.2096)
Supplement: Supplementary file 1 [file ActEsp-54-4-971-981-s1.zip › ActEsp-54-4-971-981-s1/ActEsp-54-4-971-981-1.docx]

Nagelkerke R²

Diagnosis of VIF

Normal distribution test

correlation test
